# AMP-activated protein kinase(AMPK) channel: A Global Bibliometric analysis From 2012 to 2021

**DOI:** 10.1080/19336950.2022.2049543

**Published:** 2022-03-21

**Authors:** Tianyi Lyu, Chuanxi Tian, Tianyang Tan, Jiaxuan Lyu, Kang Yan, Xirui Zhao, Ruoshui Wang, Chaoyang Zhang, Meng Liu, Yulong Wei

**Affiliations:** a School of Acupuncture-Moxibustion and Tuina, Beijing University of Chinese Medicine, Beijing, Beijing, China; b Clinical Graduate Department, Beijing University of Chinese Medicine, Beijing, Beijing, China

**Keywords:** AMPK, bibliometric analysis, metabolic diseases, cancer, stroke

## Abstract

In recent years, AMPK channel has gained considerable attention in a variety of research areas, and several academic journals have published articles on AMPK research. However, few attempts have been made to thoroughly assess the scientific output and current status systematically in this topic from a worldwide viewpoint. As a result, it is critical to adopt an appropriate visualization method to reveal the global status, future research trends, and hotspots in AMPK channel research. To investigate research hotspots/frontiers in certain domains, bibliometric analysis has been frequently utilized to determine the productivity of nations, institutions, authors, and the frequency of keywords. In this work, we used CiteSpace and VOSviewer to conduct a bibliometric analysis of AMPK channel studies from 2012 to 2021 in order to perform researchers with some directions for AMPK channel research.

## Introduction

AMP-activated protein kinase (AMPK) channel, a member of the serine/threonine (Ser/Thr) kinase family, is a sensor of energy status that regulates cellular energy homeostasis. Once activated by falling energy status, it promotes ATP production by enhancing energy-producing pathways and suppressing energy-consuming processes [1]. AMPK is well recognized for its impact on metabolism, but it also regulates mitochondrial biogenesis and disposal, autophagy, cell polarity, and cell growth and proliferation [2]. The conventional routes by which AMPK is activated by changes in AMP/ATP, ADP/ATP ratios, Ca2+ channels, or protein phosphatases that dephosphorylate Thr172 were previously well characterized [3,4].

Over the last decade, extensive research has revealed a variety of molecular pathways and physiological circumstances that govern AMPK activation. AMPK controls a variety of metabolic and physiological processes and is dysregulated in major chronic illnesses such as obesity, inflammation, diabetes, stroke, and cancer [5–7].

In recent years, AMPK channel has gained considerable attention in a variety of research areas, and several academic journals have published articles on AMPK research. However, few attempts have been made to thoroughly assess the scientific output and current status systematically in this topic from a worldwide viewpoint. As a result, it is critical to adopt an appropriate visualization method to reveal the global status, future research trends, and hotspots in AMPK channel research.

To investigate research hotspots/frontiers in certain domains, bibliometric analysis has been frequently utilized to determine the productivity of nations, institutions, authors, and the frequency of keywords [8–10]. In this work, we used CiteSpace and VOSviewer to conduct a bibliometric analysis of AMPK channel studies from 2012 to 2021 in order to perform researchers with some directions for AMPK channel research [11,12].

## Data source and search

**Figure 1.**
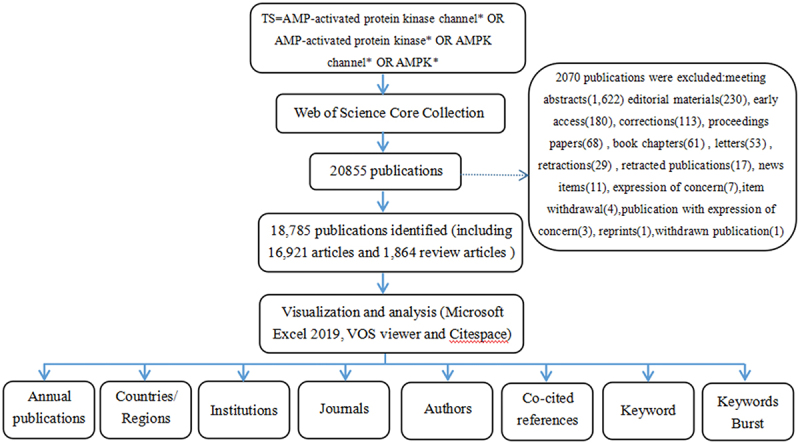
Flow chart for the analysis of AMPK channels researches.

The publications were collected from the Core Collection database of Web of Science (WoS)(http://apps.webofknowledge.com), which is widely regarded as the most authoritative database of scientific publications on a wide range of research areas. The data search was conducted on 29 November 2021. The strategy used during the search was [TS = AMP-activated protein kinase* OR AMP-activated protein kinase channel* OR AMPK* OR AMPK channel*] AND [Language = (English)] AND [Year Range = (2012–2021)]. A total of 20,855 publications were obtained, and the following documents were excluded: meeting abstracts (1,622) editorial materials (230), early access (180), corrections (113), proceedings papers (68), book chapters (61), letters (53), retractions (29), retracted publications (17), news items (11), expression of concern (7), item withdrawal (4), publication with expression of concern (3), reprints (1), withdrawn publication (1). In total, only 18,785 records (16,921 articles and 1,864 review articles) were analyzed. The data were obtained within one day to eliminate any potential variance owing to the database’s daily update. The VOS viewer 1.6.17.0 was used to identify top countries, institutions, authors and journals. The CiteSpace 5.8.R3 was used to analyze keywords, co-cited references and trends. The data analysis flow chart is shown in (Figure 1). The data for this study were taken straight from the database as secondary data, with no further animal trials. As a result, there was no need for ethical approval.

## Annual publication output

**Figure 2.**
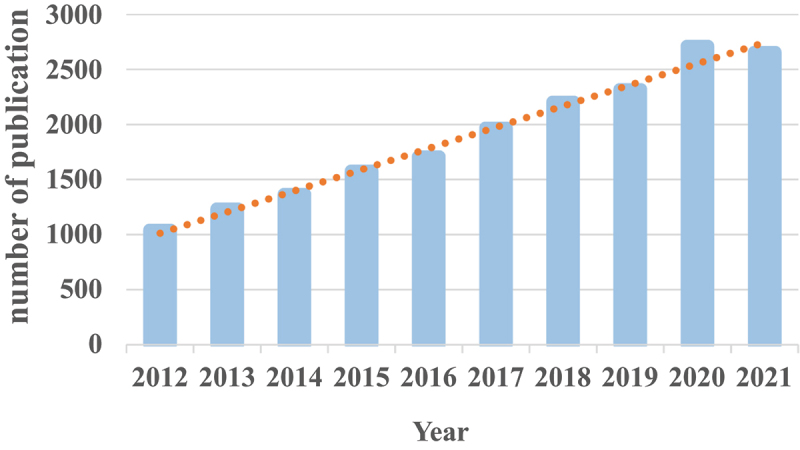
The number of annual publications on AMPK channels research from 2012 to 2021.

**Figure 3.**
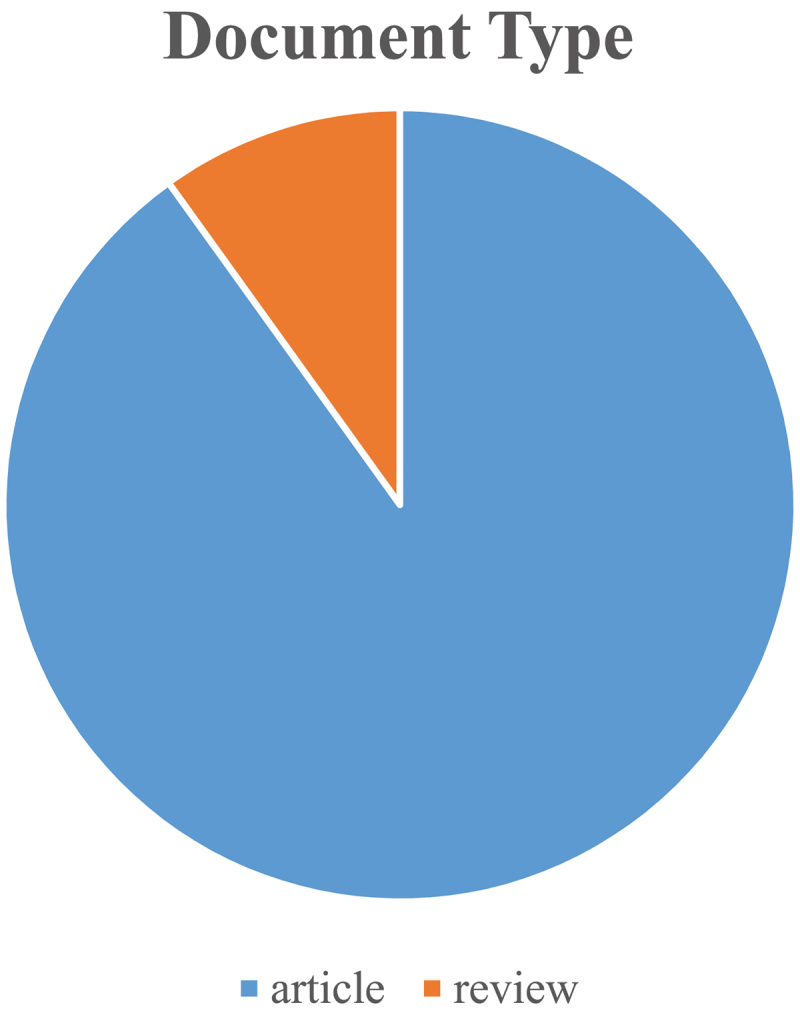
Document type. Blue represents articles and Orange represents reviews.

There were a total of 18,785 AMPK-related articles retrieved. To investigate the trends in AMPK channel research, we showed the number of articles per year in the form of a histogram. Because the annual number of published papers indicates the pace of topic knowledge and is an important metric for studying field trends [13]. As indicated in (Figure 2), the annual number of relevant publications began rapidly growing from 2012 to 2020, suggesting consistent growth and increased attention of AMPK. And, as of 29 November 2021 2,656 pieces of literature had been published in 2021. Furthermore, publications account for around 90.08% of document type (Figure 3), indicating a larger emphasis on original studies in the field of AMPK.

## Active countries and institutions

**Figure 4.**
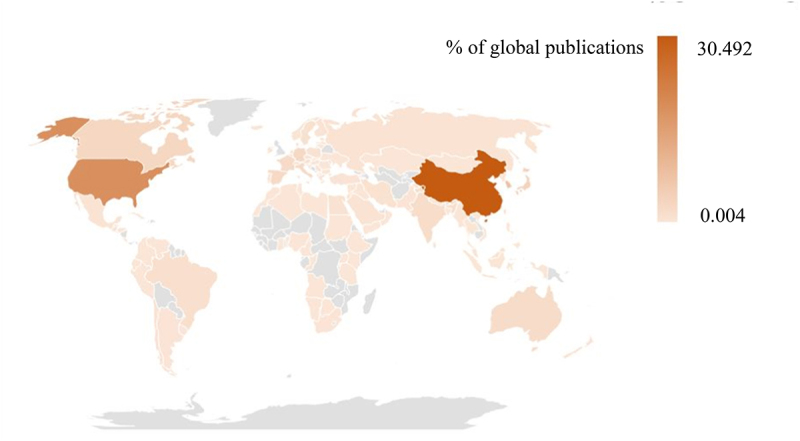
Geographical distribution map of global publications related to AMPK channels.

**Figure 5.**
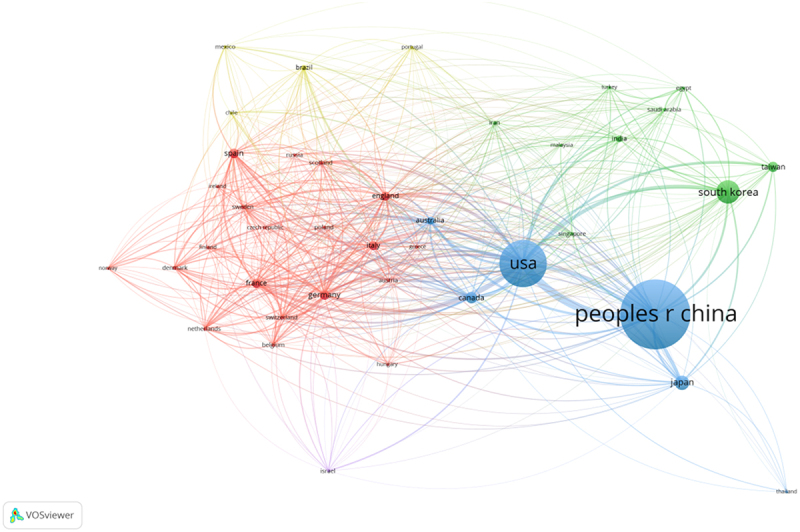
The network of countries/territories engaged in AMPK channels research.

**Figure 6.**
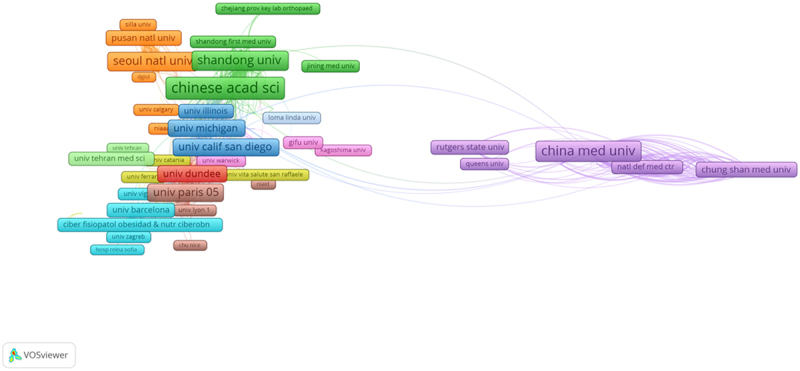
The network of institutions engaged in AMPK channel research.

Geographical distribution map of global productivity revealed that articles on AMPK channel were mostly published in North American, Asia, and European countries (Figure 4). (Table 1) shows the top 10 nations and institutions ranked by the numbers of publications on the AMPK channel. More than 9,831 research institutes in 119 countries/territories published the 18,785 pieces of literature. The co-occurrence map provides valuable information on prominent research teams and assists researchers in identifying collaborative relationships [14–16]. Countries and institutions co-occurrence maps are displayed in (Figures 5 and 6). The leading country was the CHINA, which accounted for 30.492% (7,590) of all publications, followed by USA (4641, 18.645%) and SOUTH KOREA (1904, 7.649%). (Figure 5) demonstrates that the China attached great importance to cooperation, and had close collaborations with USA, South Korea, Japan, Canada, and Australia. The Chinese Academy of Sciences was the most productive scientific research institution, producing the most number of papers on the AMPK channel (369), followed by Shanghai Jiao Tong University (324). As shown in (Figure 6), the collaboration map had 458 nodes and 8,258 links. The 458 institutions formed eight clusters with different colors. The co-occurrence map of institutions revealed that geographical location has a significant impact on scientific cooperation among institutions, and there are more collaborations among institutions in the same region (Figure 6).

**Table 1.** The top 10 countries and institutions contributed to publications on AMPK channels research

Rank	Country/ territory	Frequency	Percentage	Institution	Frequency	Percentage
1	PEOPLES R CHINA	7590	30.492%	Chinese Academy of Sciences	369	0.79%
2	USA	4641	18.645%	Shanghai Jiao Tong University	324	0.69%
3	SOUTH KOREA	1904	7.649%	China Medical University	317	0.68%
4	JAPNA	1024	4.114%	Zhejiang University	261	0.56%
5	CANADA	789	3.170%	Sun Yat-sen University	247	0.53%
6	CHINESE TAIPEI	655	2.631%	Fudan University	239	0.51%
7	GERMANY	650	2.611%	Shandong University	234	0.50%
8	SPAIN	606	2.435%	Huazhong University of Science and Technology	229	0.49%
9	ITALY	601	2.414%	Nanjing Medical University	223	0.48%
10	FRANCE	598	2.402%	Seoul National University	218	0.47%

## Active journals

**Figure 7.**
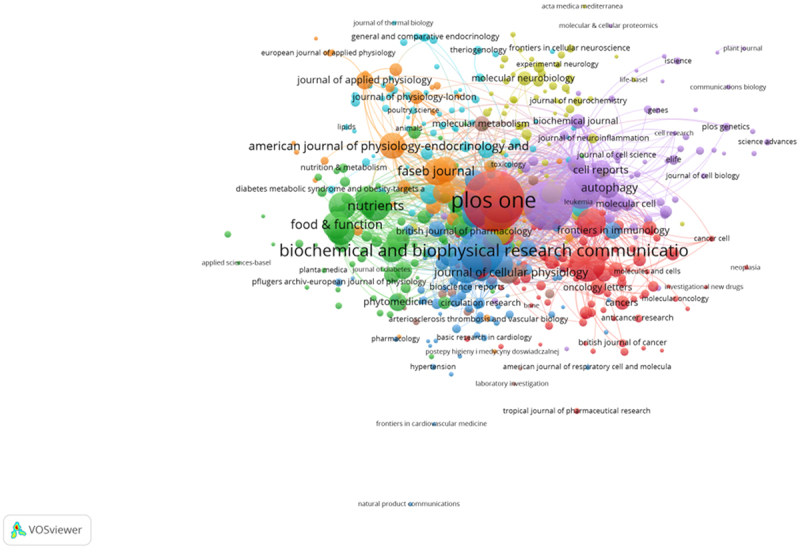
The network of journals engaged in AMPK channel.

The 18,785 literature were published in 1,879 journals. The networks shown in (Figure 7) reflect the collaboration among journals and the (Table 2) lists the top 10 journals that published articles on AMPK channel research. The Plos One had the highest number at 596 (3.173%) (IF2020 = 3.24), followed by Science Reports with 417 (2.220%) (IF2020 = 4.379), and the Journal of Biological Chemistry ranked third at 371 articles (1.975%) (IF2020 = 5.157).

**Table 2.** The top 10 journals that published articles on AMPK channels research

Rank	Journal	Frequency	% of 18,785	Total Cites	IF 2021	Country Affiliation
1	PLOS ONE	596	3.17%	16,643	3.240	US
2	SCIENTIFIC REPORTS	417	2.22%	7860	4.379	ENGLAND
3	INTERNATIONAL JOURNAL OF MOLECULAR SCIENCES	371	1.97%	5089	5.923	US
4	BIOCHEMICAL AND BIOPHYSICAL RESEARCH COMMUNICATIONS	354	1.88%	5324	3.575	US
5	ONCOTARGET	243	1.29%	5905	/	US
6	JOURNAL OF BIOLOGICAL CHEMISTRY	242	1.29%	8076	5.157	US
7	FRONTIERS IN PHARMACOLOGY	214	1.14%	2144	5.810	SWITZERLAND
8	NUTRIENTS	177	0.94%	2432	5.717	SWITZERLAND
9	BIOMEDICINE&PHARMACOTHERAPY	171	0.91%	2003	6.529	FRANCE
10	CELL DEATH&DISEASE	164	0.87%	4155	8.469	ENGLAND

## Active authors

**Figure 8.**
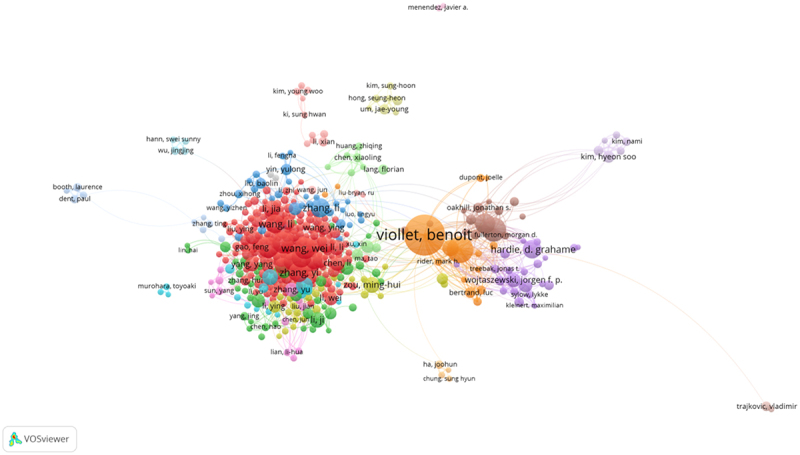
The network of authors contributed to AMPK channels research.

Author co-occurrence map can provide information on influential research groups and potential collaborators. It can assist researchers to locating potential collaborators [14,15]. In the 18,785 publications related to AMPK channel research, a total of approximately 86,270 authors were obtained. The networks depicted in (Figure 8) represent author collaboration, and the top 10 active authors are listed in (Table 3). Viollet Bennoit contributed the most papers (144 publications, 0.052%), primarily on different AMPK-dependent and AMPK-independent mechanisms underlying diabetes [17,18], followed by Foretz Marc and Steinberg. Gregory R. with 83 and 74 publications, respectively. There was an active collaboration among the productive authors.

**Table 3.** The top 10 active authors in AMPK channels research

Rank	Author	Frequency	Percentage
1	Viollet Bennoit	144	0.052%
2	Foretz Marc	83	0.030%
3	Steinberg Gregory R.	74	0.027%
4	Wang, Wei	65	0.024%
5	Wang, Jin	63	0.023%
6	Lopez Miguel	61	0.022%
7	Li, Yan	61	0.022%
8	Kemp, Bruce E.	60	0.022%
9	Liu, Wei	58	0.021%
10	Zhang, Yi	57	0.021%

## Co-cited reference

**Figure 9.**
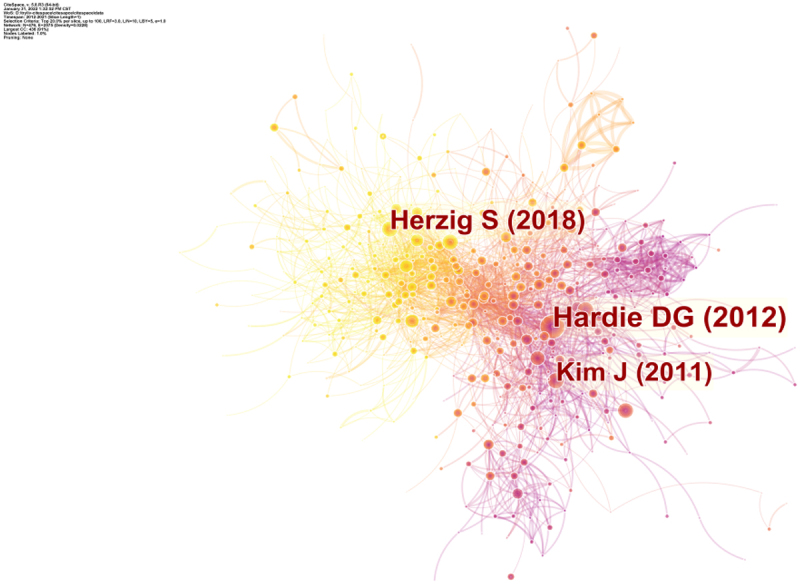
The analysis of Co-cited references: Co-cited network of referenced from publications on AMPK channels.

A total of 18,785 publications were visualized and analyzed using CiteSpace 5.8.R3 from 2012 to 2021, with a time slice of 1 chosen for the analysis of co-cited references. The network of co-cited references on AMPK channel with highest centrality and citation counts is presented in (Figure 9). The most cited references were examined to determine the key knowledge base in the field. The top 10 most frequently co-cited references are summarized in (Table 4). These articles laid the foundation for further research into the structure and mechanism of the AMPK channel. Furthermore, these articles provided a theoretical basis for the study of AMPK channel.

**Table 4.** The top 10 co-cited references in AMPK channels research

Rank	Frequency	Author	Year	Source	Co-cited Reference
1	806	Hardie DG	2012	NAT REV MOL CELL BIO	AMPK: a nutrient and energy sensor that maintains energy homeostasis
2	517	Herzig S	2018	NAT REV MOL CELL BIO	AMPK: guardian of metabolism and mitochondrial homeostasis
3	438	Kim J	2011	NAT CELL BIOL	AMPK and mTOR regulate autophagy through direct phosphorylation of Ulk1
4	364	Garcia D	2017	MOL CELL	AMPK: Mechanisms of Cellular Energy Sensing and Restoration of Metabolic Balance
5	355	Mihaylova MM	2011	NAT CELL BIOL	The AMPK signaling pathway coordinates cell growth, autophagy and metabolism
6	323	Hardie DG	2011	GENE DEV	AMP-activated protein kinase – an energy sensor that regulates all aspects of cell function
7	310	Hardie DG	2016	TRENDS CELL BIOL	AMPK: An Energy-Sensing Pathway with Multiple Inputs and Outputs
8	288	Egan DF	2011	SCIENCE	Phosphorylation of ULK1 (hATG1) by AMP-Activated Protein Kinase Connects Energy Sensing to Mitophagy
9	258	Jeon SM	2016	EXP MOL MED	Regulation and function of AMPK in physiology and diseases
10	242	Xiao B	2011	NATURE	Structure of mammalian AMPK and its regulation by ADP

**Table 5.** Top 20 keywords in terms of frequency in AMPK channels

Rank	Keywords	Frequency	Rank	Keywords	Frequency
1	ampk	6716	11	insulin-resistance	1643
2	activated protein-kinase	4250	12	inhibition	1612
3	expression	3061	13	inflammation	1605
4	autophagy	2741	14	obesity	1454
5	oxidative stress	2506	15	skeletal-muscle	1403
6	apoptosis	2372	16	protein-kinase	1335
7	metabolism	2239	17	cells	1322
8	activation	2196	18	pathway	1321
9	phosphorylation	1968	19	growth	1281
10	metformin	1643	20	gene-expression	1123

**Figure 10.**
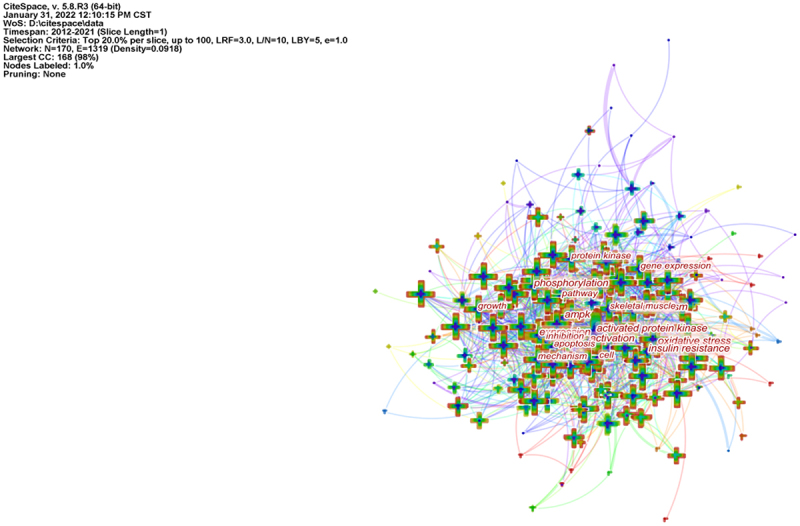
The analysis of keywords in AMPK channels research.

The first highly co-cited article was “AMPK: a nutrient and energy sensor that maintains energy homeostasis” (806 citation rate), in which Hardie DG revealed that AMPK regulated metabolic energy balance at the whole-body and intracellular levels via the ATP processes [19]. Other three co-cited references were published in Nature, Science, and Nature Reviews molecular cell biology: Xiao B developed a model that explained how the energy status of a cell regulated AMPK activity from biochemical and structural data [20]. Egan DF identified a conserved biochemical mechanism that coupled nutrient status with autophagy and cell survival [21]. In 2018, Herzig S discussed how AMPK functions as a central mediator of the cellular response to energetic stress and mitochondrial insults, as well as how it coordinated multiple features of autophagy and mitochondrial biology [1].

## Keyword co-occurrence

Keywords were extracted from the 18,785 publications, which is the most important part of the research (Figure 10 and Table 5). Keywords co-occurrence analysis provides a reasonable description of research hotspots and burst keywords can represent research frontiers over a period of time [22].

CiteSpace 5.8.R3 was used to construct an overlay visualization map of keyword co-occurrence and to identify the top 20 keywords in AMPK channel research from 2012 to 2021, in terms of frequency. The top keywords were “ampk,” “activated protein-kinase,” “expression,” “autophagy,” “oxidative stress,” “apoptosis,” “metabolism,” “activation,” “phosphorylation,” “metformin,” “insulin-resistance,” “inhibition,” “inflammation,” “obesity,” “skeletal-muscle,” “protein-kinase,” “cells,” “pathway,” “growth,” “gene-expression.” Therefore, research hotspots can be summarized in the following aspect.

Regulation of AMPK in metabolic diseases

AMPK channel involves the regulation of many important physiological and pathological processes and has received a lot of attention as a potential target for treating diseases associated with metabolic perturbation. This included obesity, Type-2 diabetes, liver diseases and so on.

- AMPK and Type-2 diabetes

Type-2 diabetes is a metabolic syndrome caused by insulin resistance that induces hyperglycemia, hyperinsulinemia and hyperlipidemia. Intensive research has shown that prolonged exposure to excessive nutrients is one of the critical risk factors of insulin resistance [23]. High FFAs can drive insulin resistance through DAG accumulation and PKC activation, which impairs insulin signaling by phosphorylating (IRS)-1/2. These pathways are associated with reduced inhibitory phosphorylation of AMPK channel [24]. Indeed, metformin, an indirect activator of AMPK, is the most frequently prescribed antidiabetic drug for type-2 diabetic patients [25]. Therefore, AMPK-activating agents would be beneficial for both preventing and treating patients with type-2 diabetes.

- AMPK and obesity

Studies accrued over the last decade have demonstrated an unequivocally key role of hypothalamic AMPK in the regulation of both parts of the energy balance equation, i.e. feeding and energy expenditure [26]. Activation of AMPK in hypothalamus seems to give the best outcome in treatment for obesity. The use of nanoparticle or exosome approaches might be an option. Another alternative might be optogenetic modulation of hypothalamic AMPK neurons, which has already been elegantly achieved in rodents [27]. However, the most relevant issue to address is that of the long-term consequences of targeting AMPK in the brain [28]. A substantial amount of work will be necessary to understand the molecular and neural mechanisms upstream and downstream of central AMPK fully.

Regulation of AMPK in cancer

Tumor suppressor LKB1 functions as an upstream kinase, and mTORC1 functions as a downstream effector of AMPK [29]. Therefore, AMPK activation would be a promising therapeutic strategy because it inhibits mTORC1. A theory of multistep carcinogenesis indicates that the tumor initiation stage, which is characterized by the introduction of DNA mutations in normal chromosomes, favors the formation of a stressful and proinflammatory environment for inducing genetic mutations. Inactivation of the LKB1-AMPK pathway during this stage may facilitate both cell growth and proliferation by activating mTORC1 and anabolic pathways, and by introducing genetic mutations through the augmentation of oxidative stress and proinflammatory response [30].

Regulation of AMPK in cerebral diseases

It is generally acknowledged that AMPK is a crucial cellular energy sensor and regulates metabolic energy balance at the whole-body level [31]. Meanwhile, neurons are the most metabolically demanding cells in human body. Neurons utilize only glucose and poorly store energy, which makes neurons more vulnerable to cerebral ischemia that might cause irreversible injury to the cerebrum [32]. Under harmful stimuli, neurons can be activated and have a higher expression of AMPK for glucose uptake and utilization. Therefore, sufficient and stable AMPK content is necessary to maintain the life and activity of neurons. Moreover, a number of studies have indicated that AMPK is able to protect against ischemic stroke, hemorrhage stroke and NDD, suggesting that AMPK is a protective regulator in cerebral diseases [33,34].

## Keyword co-occurrence and burst

To investigate research hotspots, frontiers, and emerging trends over time, keywords were identified and analyzed using strong citation bursts (Table 6). The red line in (Table 6) indicates the time span during which the burst keyword appears [35]. The fact that the citation burst time of keywords such as “fission” (2018–2021, 9.16), “stroke” (2018–2021, 9.12), “ampk pathway” (2019–2021,10.03), “acute kidney injury” (2019–2021, 9.36), and “axi” (2019–2021,9.3) has continued to 2021 and is still ongoing demonstrates that these directions have great potential.

**Table 6.** Top 30 keywords with the strongest citation bursts

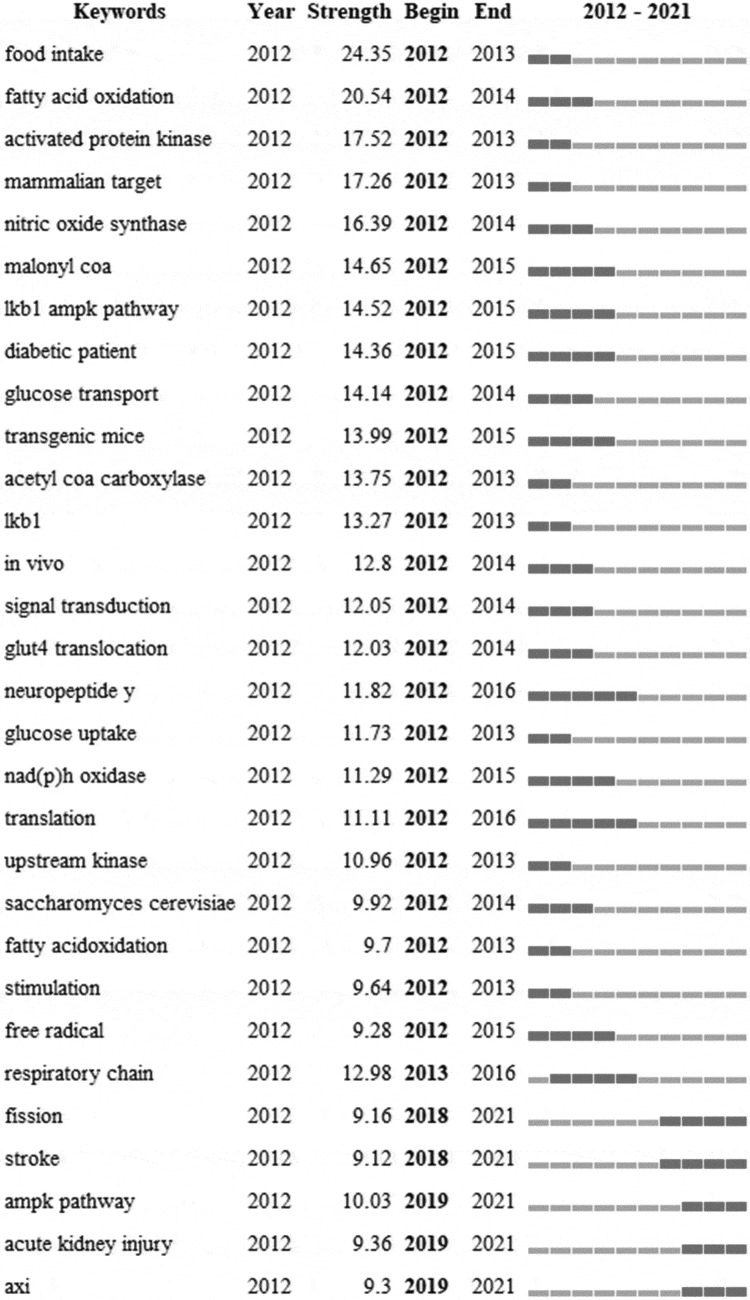

## Conclusions

Bibliometric and visual analysis were used to study the characteristics of AMPK channel research results from 2012 to 2021 using the WOSCC database. The number of publications on AMPK channels has remained constant at more than 1,000 per year. Based on current global trends, there will be a dramatic increase in the number of publications on AMPK research. China is the leading country in AMPK channel research. “Oxidative stress,” “insulin resistance,” “apoptosis,” and “skeletal muscle” were the hotspots of AMPK channel research. More attention will be paid to “stroke,” “acute kidney injury,” and “fission,” which are likely to be the next popular topics in AMPK channel research.

To the best of our knowledge, this study is the first bibliometric analysis focusing on AMPK channel trends. The data downloaded from WoS covered the vast majority of articles in the field of AMPK channel research and the data analysis was relatively objective and comprehensive, clearly showing the status of AMPK channel.

Based on presented results, reseachers working in the field of AMPK channel can identify potential collaborations, locate research hotspots and forecast research frontiers. Organizations can refer to this article as a reference when deciding whether or not to provide repeat funding to a given research team.
